# Calcium mediated functional interplay between myocardial cells upon laser-induced single-cell injury: an in vitro study of cardiac cell death signaling mechanisms

**DOI:** 10.1186/s12964-020-00689-5

**Published:** 2020-12-28

**Authors:** Krishna Chander Sridhar, Nils Hersch, Georg Dreissen, Rudolf Merkel, Bernd Hoffmann

**Affiliations:** Institute of Biological Information Processing, IBI-2: Mechanobiology, Forschungszentrum Jülich, 52425 Jülich, Germany

**Keywords:** Myocardial infarction, Cardiomyocyte, Cardiac fibroblast, Calcium transport, Gap junction, Laser ablation, Induced cell death

## Abstract

**Background:**

The electromechanical function of myocardial tissue depends on the intercellular communication between cardiomyocytes (CMs) as well as their crosstalk with other cell types. Cell injury, and subsequent death trigger inflammation as in myocardial infarction (MI) resulting in myocardial remodeling. Although mechanisms underlying myocardial cell death have been studied so far, the signaling events following single cell death and spontaneous response of connected cells in the myocardial tissue is still barely understood.

**Methods:**

Here, we investigated the effect of laser-induced single cell death on Calcium (Ca^2+^) concentrations and transport in myocardial cell clusters in vitro. Spatial and temporal changes in intracellular Ca^2+^ concentrations [Ca^2+^]_i_ were studied using a fluorescent calcium indicator, Fluo-4AM. Spontaneous signaling events following cell death were studied in rat embryonic cardiomyocytes and non-myocytes using separate cell culture systems.

**Results:**

Cell death triggered spontaneous increase in intracellular Ca^2+^ levels ([Ca^2+^]_i_) of surrounding cells. The spread of the observed propagating Ca^2+^ signal was slow and sustained in myocytes while it was rapid and transient in fibroblasts (Fbs). Further, sustained high Ca^2+^ levels temporarily impaired the contractility in CMs. The cell-type specific effect of ablation was confirmed using separate cultures of CMs and Fbs. Comparing Ca^2+^ propagation speed in myocytes and fibroblasts, we argue for a diffusion-driven Ca^2+^ propagation in myocytes, but not in fibroblasts. Radial and sequential Ca^2+^ diffusion across the CMs through cell–cell contacts and presence of Cx43-based intercellular junctions indicated a gap junction flow of Ca^2+^.

**Conclusions:**

These findings illustrate the spontaneous Ca^2+^-mediated functional interplay in myocardial cell clusters upon mechanical injury and, further, the difference in Ca^2+^ signaling in cardiomyocytes and fibroblasts.

**Video Abstract**

## Background

Myocardial tissue function is orchestrated by a network of cardiomyocytes (CMs), electrically and mechanically connected to each other to effect coordinated heart muscle contraction. The electrical connectivity in CMs is established through the conduction of depolarization current (action potential) from pacemaker cells [1] while the mechanical connectivity is achieved by the repeating units of myofibrils [2] connected at intercellular junctions [3]. Mechanical contraction in CMs is triggered by electrical activation via an intracellular calcium-dependent process known as excitation–contraction coupling [4]. Myocardial infarction (MI) results in large-scale death of CMs in the myocardium while other cardiomyopathies result in sporadic CM death [5]. Loss of CMs leads to activation of inflammatory cascades and scarring that results in remodeled myocardium [6] with altered electrical and mechanical properties [7]. Several studies so far have focused on pathways leading to CMs death and subsequent myocardial remodeling that leads to structural and functional imbalance in the myocardial tissue [8, 9]. However, the impacts of single CM death on connected cells and the signals accompanying cell death are yet to be thoroughly investigated although myocardial cell injury is accompanied with various diseases like ischemia, reperfusion or infarction [10, 11]. Therefore, exploring the impact of cell death on connected cells could provide valuable knowledge on cell death signals and functional connectivity of cells in myocardial pathologies.

Though cardiomyocytes make up about two-third of the heart by volume, non-myocytes such as fibroblasts (Fbs) and vascular cells outnumber them [12] owing to their relatively smaller size and higher proliferative potential unlike the myocytes with limited regenerative potential [13]. Fibroblasts form a functionally significant sub-population of non-myocytes [12, 14] as they provide structural support to the myocardium by regulating the synthesis and degradation of the extracellular matrix (ECM) [15]. Fibroblast-proliferation associated with aging and CM death adversely changes tissue-stiffness and electrical behavior of the myocardium [7, 16]. Further, increasing evidence of the critical functions of fibroblasts in normal and diseased myocardium point out their importance for cardiac models [17, 18]. Electrical coupling between myocytes and fibroblasts has been reported indicating that Fbs are involved in conduction of electrical impulses [19, 20]. Recently, well defined in vitro models of myocardial cells have been developed to study functional connectivity in homogeneous and heterogeneous cell pairs [21, 22]. Yet, the nature of intercellular signaling between CMs and Fbs and its implications in myocardial tissue remains elusive.

In our present study we demonstrate the effect of single-cell death induced by controlled laser-ablation of cultured myocardial cells (CMs and Fbs). This technique has been used successfully before both in vitro and in vivo to describe wounding pattern and stress response in cells and tissues [23–25]. Ca^2+^ has been used in functional studies as it regulates vital cellular functions in mammalian cells [26]. Especially in myocardial cells, study of Ca^2+^ dynamics is highly useful as: (1) Ca^2+^ sparks are fundamental for CM contraction [27], (2) Ca^2+^ serves as a second messenger in critical cellular processes such as transcription and apoptosis [28], and (3) real-time intracellular Ca^2+^ changes and waves indicate cellular activity and intercellular connectivity in CMs [29]. Moreover, in the context of our study, the difference in the intracellular Ca^2+^ levels (sparks) could be used to distinguish myocytes from non-myocytes. Here we describe the spontaneous response of myocardial cells (CMs and Fbs) in mixed and enriched cultures to mechanical damage induced by laser ablation. Following a fluorescent Ca^2+^ indicator, we qualitatively studied the changes in [Ca^2+^]_i_ in cultured myocardial cells upon single cell injury. Based on the spatial and temporal [Ca^2+^]_i_ changes, we describe the functional connectivity and Ca^2+^ signal propagation in both myocytes and fibroblasts.

## Materials and methods

### Primary cell culture from embryonic rat hearts

Primary cardiac cell culture was obtained from embryonic rat (18–19 days post fertilization) hearts as described in Hersch et al.[30] (Animal testing license: 81-02.04.2018.A90, LANUV NRW, Germany). Cells were maintained in F10 Ham’s medium (Sigma-Aldrich, St. Louis, MO) supplemented with 10% fetal bovine serum, a 1/100 dilution of an antibiotic solution (10,000 units penicillin and 10 mg/ml streptomycin in 0.9% NaCl, (Sigma-Aldrich)) and a 1/200 dilution of ITS liquid media supplement containing insulin (1 mg/ml), transferrin (0.55 mg/ml), and sodium selenite (0.5 mg/ml) in Earle’s balanced salt solution (EBSS, Sigma-Aldrich) [30]. Isolated cells were cultured on petri dishes fitted with glass cover slips (1.5# high-precision cover slips with a thickness of 170 ± 5 µm, Paul Marienfeld, Lauda-Königshofen, Germany) at the bottom for confocal imaging. Prior to seeding of cells, substrates were coated with 10 µg/ml human fibronectin (Corning, Tewksbury, MA) in Phosphate buffered saline (PBS) and incubated at 37 °C for 20 min. About 100,000 cells were cultured in a humidified atmosphere with 5% CO_2_ at 37 °C. Cultured cells contracted synchronously in clusters from 48–72 h and were used for subsequent experiments.

### Cell separation using fusogenic liposomes

Myocytes were separated from remaining myocardial cells using fusion based biotin-labelling of cells for subsequent cell-separation using magnetic anti-biotin microbeads as described in Hersch et al.[30]. Fusogenic liposomes (FLs) containing lipids 1,2-dioleoyl-sn-glycero-3-phosphoethanolamine (DOPE), 1,2-dioleoyl-3-trimethylammonium-propane (chloride salt, DOTAP), 1,2-dioleoyl-sn-glycero-3-phosphoethanolamine-*N*-(cap biotinyl) (sodium salt, biotin-DOPE) from Avanti Polar Lipids, Inc., (Alabaster, AL) and 1,10-dioctadecyl-3,3,30,30-tetramethylindotricarbocyanine iodide (DiR, Life technologies, Eugene, OR) were prepared in the molar ratio 1/1/0.1/0.05. For fusion, 1–2 × 10^6^ freshly isolated cells were incubated with 20 µl biotin-FLs in 1 ml Dulbecco’s modified eagle medium (DMEM, 11960044, Thermo Fisher, Waltham, MA) for 2 min at room temperature. After biotinylation, cells were centrifuged and incubated at 4 °C for 20 min with 20 µl anti-biotin magnetic microbeads (130-090-485, Miltenyi Biotech, Bergisch Gladbach, Germany) diluted 1:5 in cell culture medium. The resulting suspension was further diluted with culture medium, centrifuged, and the pellet was resuspended in culture medium. The cell suspension was introduced into the magnetic separation column (MiniMACS, Miltenyi Biotec). Due to high fusion efficiency for non-myocyte cells and low efficiency for myocytes, the flow-through was enriched with myocytes while all other cells were retained in the magnetic column. Removing the magnetic field freed the attached cells and enabled cell collection. The cells were seeded separately to culture dishes and cultured as described above.

### Live cell calcium imaging in cultured myocardial cells

Cultured cells were washed with pre-warmed culture medium and calcium indicator Fluo-4AM (Molecular Probes, Eugene, OR) was added to cells at a final concentration of 5 µM in culture medium. The cells were incubated for 20 min at 37 °C. Extracellular fluorophore was removed by washing with warm medium. For confocal microscopy (LSM880, Carl Zeiss, Jena, Germany) the fluorophore was excited at 488 nm and detected with a 490–550 nm bandpass filter. Continuous imaging at 160 ms per frame was used to record calcium waves using a 20 × Plan-Apochromat objective (NA 0.8) and a pixel size of 1.66 µm.

### Laser ablation of target cells

Clusters of contracting cardiomyocytes exhibiting rhythmic Ca^2+^ sparks were chosen. A pulsed UV-laser, λ = 355 nm (RAPP optoelectronics, Wedel, Germany) was used for targeted killing of single chosen cells in a cluster. Before ablation the laser position was calibrated in x, y and z directions. The effective laser power, beam width, and number of iterations were chosen. Single cells of a cluster were irradiated with laser intensities between 2 and 5% of total laser output. Cell death was observed from membrane disruption and cell retraction in phase contrast imaging and from spontaneous loss of Ca^2+^ fluorescence in these cells.

### Immunocytochemistry

Cultured cells were fixed in paraformaldehyde (3.7%) in cytoskeletal buffer (CB) – 150 mM NaCl; 5 mM MgCl_2_; 5 mM ethylene glycol-bis(2-aminoethylether)-*N*,*N*,*N*′,*N*′-tetraacetic acid (EGTA); 5 mM glucose; 10 mM 2-(4-morpholino)ethanesulfonic acid (MES); pH 6.1 [30]. After fixation, the cells were treated in 100 mM glycine-CB solution for 20 min at RT. Membrane permeabilization was performed using 0.5% Triton-X-100 (Sigma-Aldrich) solution in CB for 10 min. Unspecific labeling was reduced by incubating cells with blocking solution (5% solution of dry milk (Carl Roth, Karlsruhe, Germany) in CB) for 90 min. Cells were then incubated with a 1:100 dilution (in 1% solution of dry milk in CB) of primary antibodies, mouse sarcomeric anti-α-actinin, clone EA-53 monoclonal antibody (A7811, Sigma-Aldrich) and rabbit polyclonal anti-connexin43 (AB1728, Merck, Darmstadt, Germany) overnight at 4 °C. For labelling only myofilaments (sarcomeres) to distinguish cell culture systems, Cy3-conjugated goat anti-mouse (115-165-006, Jackson Immunoresearch, Baltimore Pike, PA) secondary antibody was used. For double staining of sarcomeres and connexin43, Alexa 488 goat anti-mouse (A11001, Invitrogen, Carlsbad, CA) and Cy3-conjugated goat anti-rabbit (111-166-046, Jackson Immunoresearch) secondary antibodies were used. All secondary antibodies were diluted 1:200 in CB containing 1% dry milk. For labeling F-actin, Phalloidin-Atto 633 (68825, Sigma-Aldrich) was added to the cells at a dilution of 1:300 in parallel to the secondary antibodies and incubated at RT for 2 h. For nuclear staining, NucBlue (R37605, Molecular Probes) was added to the cells after secondary antibody incubation as per manufacturer’s guidelines. The samples were stored in CB for confocal microscopy (LSM880, Carl Zeiss). Samples were excited with 405 nm, 488 nm, 561 nm and 633 nm laser lines and the emission was detected with 415–475 nm, 490–535 nm, 550–600 nm and 634–680 nm bandpass filters. Tile scanning was performed using a motorized scanning stage (centered grid scanning) to obtain overview images of samples around the existing stage position. An EC Plan-Neoflaur 40 × oil objective (NA 1.3) was used to image an area of 1.55 mm × 1.55 mm, divided into 64 (8 × 8) tiles with 10% overlap of the tiles (rectangular grid tiling mode). The LSM based built-in autofocus (ZEN, Carl Zeiss) was used to compensate for the z-drift and was set for every four tiles. The autofocus was set to fluorescence mode which is ideally suited for imaging monolayers with stained nuclei where the nuclei provided an ideal reference plane for imaging. The tile images were stitched together using the built-in online stitching tool (ZEN, Carl Zeiss; threshold value of 0.7) during acquisition to obtain a single overview image of the culture.

### Analysis of intracellular Ca^2+^ changes

Ablation of cells induced specific Ca^2+^ signals in their neighborhood. These consist of a propagating Ca^2+^ wave and, for myocytes, a temporary interruption of regular beating. To quantify these changes we implemented algorithms in Matlab (R2017) to determine the distance-dependent fluorescence intensity of the Ca^2+^ indicator Fluo-4AM. Based on these curves we determined the propagation speed of the slow Ca^2+^ wave induced by ablation and, for cardiomyocytes, how long it took until these cells resumed beating.

#### Determination of intensities from Ca^2+^ imaging movies

**Fig. 1:**
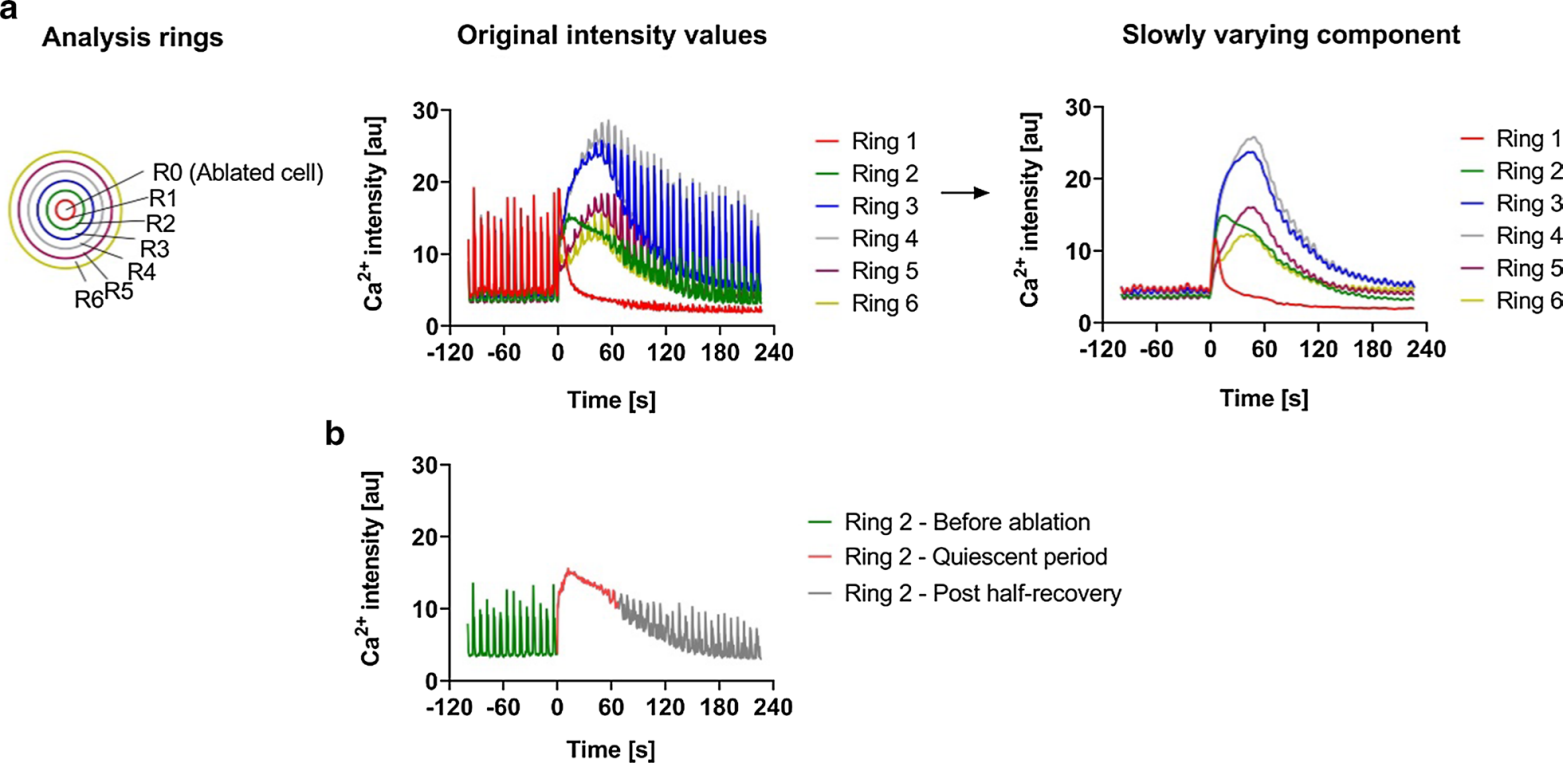
Fluorescence intensity-changes in cells over time: **a** Ca^2+^-induced fluorescence intensity-changes over the time course of ablation *(center,* grey values*)* in each of the analysis rings (*left*). The corresponding slowly-varying signal component of fluorescence intensity (*right,* grey values) represents the local minima value for every intensity peak in the original intensity plots, plotted against time. Ablation occurred at 0 s. The numbering of rings starts from the central ring containing the ablated cell (shown as R1, *left*) and then successively until the last selected ring (R6). **b** Ca^2+^ changes in cells in the second analysis ring (R2) from the plots illustrated above during pre-ablation, quiescent period following ablation, and post-half-recovery periods, indicated in different color coding, based on the half-recovery time is shown as an example of the algorithm described above

In a first step the ablation point was marked manually. Around the ablation point concentric rings of 20 µm width were generated as shown in Fig. 1a (left). At each time point average intensities of all rings were calculated.

#### Propagation speed of the Ca^2+^ wave

For each ring we determined the time point of maximum intensity and calculated the speed as ratio of the radius of the ring and the delay between ablation and maximum intensity. While this could be done directly on the measured curves for non-myocytes, the rapid transients of beating myocytes necessitated a further processing step to extract the much slower signal of the calcium wave (see Fig. 1a). To this end we used an algorithm proposed by Eilers and Boelens [31]. It consists of a Whittacker smoother of second order (with penalty factor λ set to 10) [32] combined with asymmetric least square fitting (with asymmetry parameter Φ set to 0.015) [33]. This algorithm produced a faithful representation of the slowly varying signal while short calcium spikes were effectively suppressed. Only rings with an at least two-fold calcium increase were analyzed. Moreover, in some cases signals from rings influenced by clearly unconnected cells had to be discarded.

#### Determination of Ca^2+^ fold-change after ablation

Ca^2+^ fold-change in myocytes and non-myocytes following ablation was determined based on the slowly varying Ca^2+^ signal intensities for each analysis ring before and after ablation. The average Ca^2+^ intensity before ablation was calculated for each ring as a first step. The maximum Ca^2+^ intensity after ablation was then determined. The ratio of maximum Ca^2+^ signal intensity after ablation to the average intensity before ablation was calculated for each of the analysis rings for all cultures. This represented the Ca^2+^ fold-change after ablation for each of the analysis rings.

#### Determination of the quiescent period

Because myocytes resumed beating in a gradual way, we developed an algorithm to determine the period during which the residual beats remained below 50% of the pre-ablation period. In this algorithm, the measured intensity traces were first high-pass filtered in Fourier space (hard cut-off at 0.5 Hz). Instantaneous amplitudes were determined as the difference between the highest and the lowest signal intensity in a 10 s interval centered on any given time point. The resulting discontinuous signal was smoothed three times with a moving average in a centered window of width 10 s (Matlab routine movmean). In this smoothed trace intensity was averaged over the full pre-ablation period and the time interval between ablation and first signal increase above 50% of this value was taken as the quiescent period. An example of the different time periods of Ca^2+^ imaging experiments (pre-ablation, quiescent and post-half-recovery) provided by the algorithm is shown in Fig. 1b for one of the analysis rings (Ring 2) selected from the original intensity plot in Fig. 1a.

#### Determination of Ca^2+^ full recovery times in myocytes and non-myocytes after ablation

The time-point at which Ca^2+^ intensities completely recovered to the initial levels before ablation was determined using Python (Python version 3.8.3 for Windows). To detect the first time point where the signal reaches the initial value again, a Python script was used. First, for each ring the signal before ablation was averaged and defined as reference value. Then, the signal after ablation was averaged using a sliding window with a block size of 20 time points (3.2 s). The first time point where a block of the sliding window was below the reference value was defined as time point of recovery.

### Statistical analysis

Multiple t-tests by Holm-Šídák method were performed using GraphPad Prism version 8.4.3 for Windows, GraphPad Software, La Jolla California USA, www.graphpad.com. The significance levels were set to 5% (α = 0.05) for all the analyses. P-value from the statistical tests were indicated by * for P ≤ 0.05, ** for P ≤ 0.01, *** for P ≤ 0.001 and **** for P ≤ 0.0001.

## Results

### Cell culture systems from embryonic rat hearts using fusogenic liposomes

**Fig. 2:**
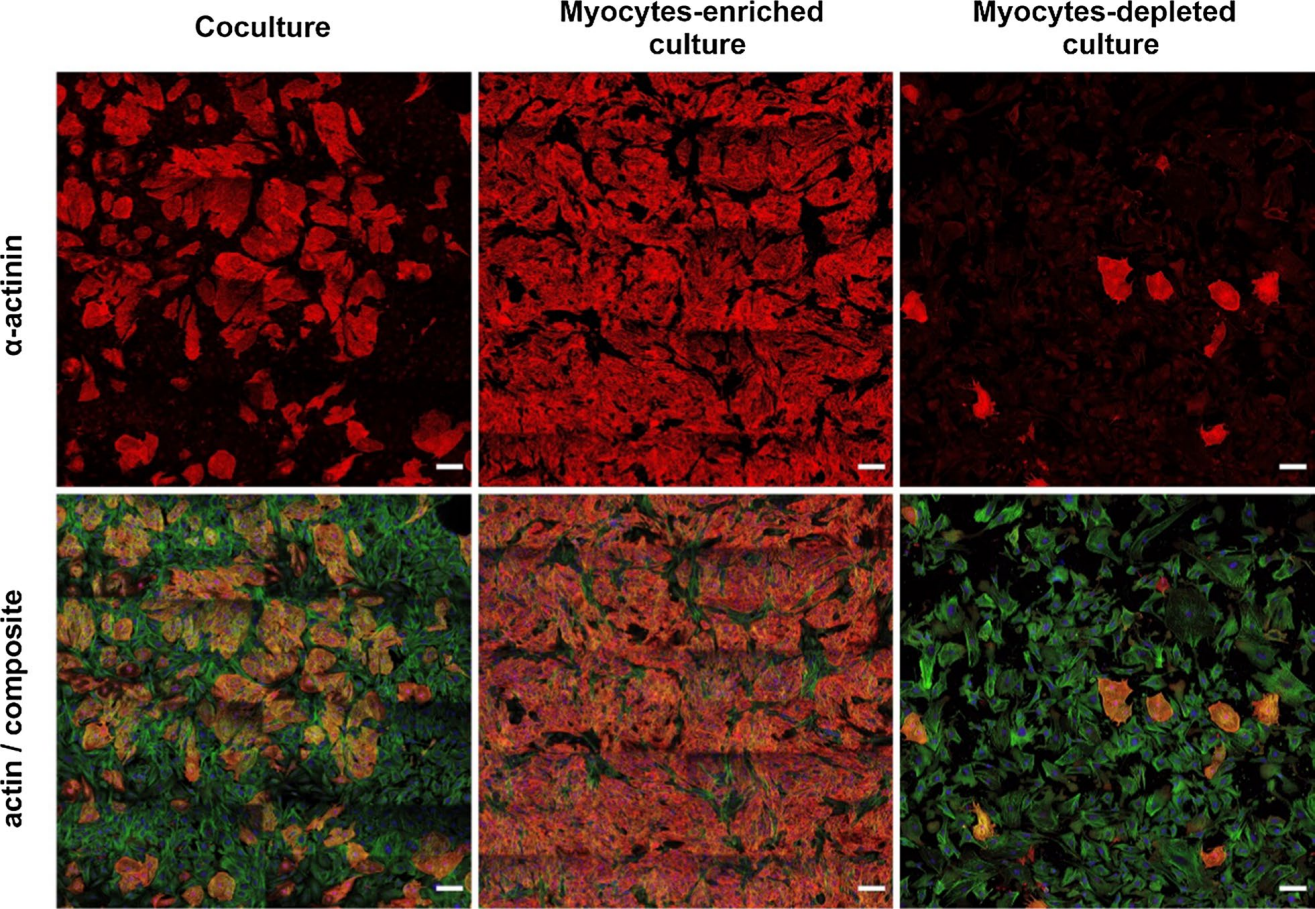
Primary cell culture from embryonic rat hearts: freshly isolated myocardial cells were either analyzed as cocultures or further purified as described before [30] to result in either myocyte-enriched or myocyte-depleted cultures. Overview images of day 3 cultures are shown. Cardiomyocytes were distinguished from non-myocyte cells by presence of the sarcomere-specific α-actinin (red). Actin was labelled in green and nuclei in blue. All images represent stitched tile images to clarify the quality of the purification in the overview. Scale bars: 100 µm

Three cell culture systems were prepared based on myocyte and non-myocyte cell population namely the coculture, myocytes-enriched culture and myocyte-depleted culture system as shown in Fig. 2. Following the procedure described before [30], efficient myocyte purification could be performed. Remaining cells were accordingly significantly reduced by myocytes and used as myocyte-depleted culture. For simplicity reasons all non-myocyte cells are named as fibroblasts in this work.

### Induced single-cell death results in increased intracellular calcium concentration ([Ca^2+^]_i_) in surrounding cells of cocultures

**Fig. 3:**
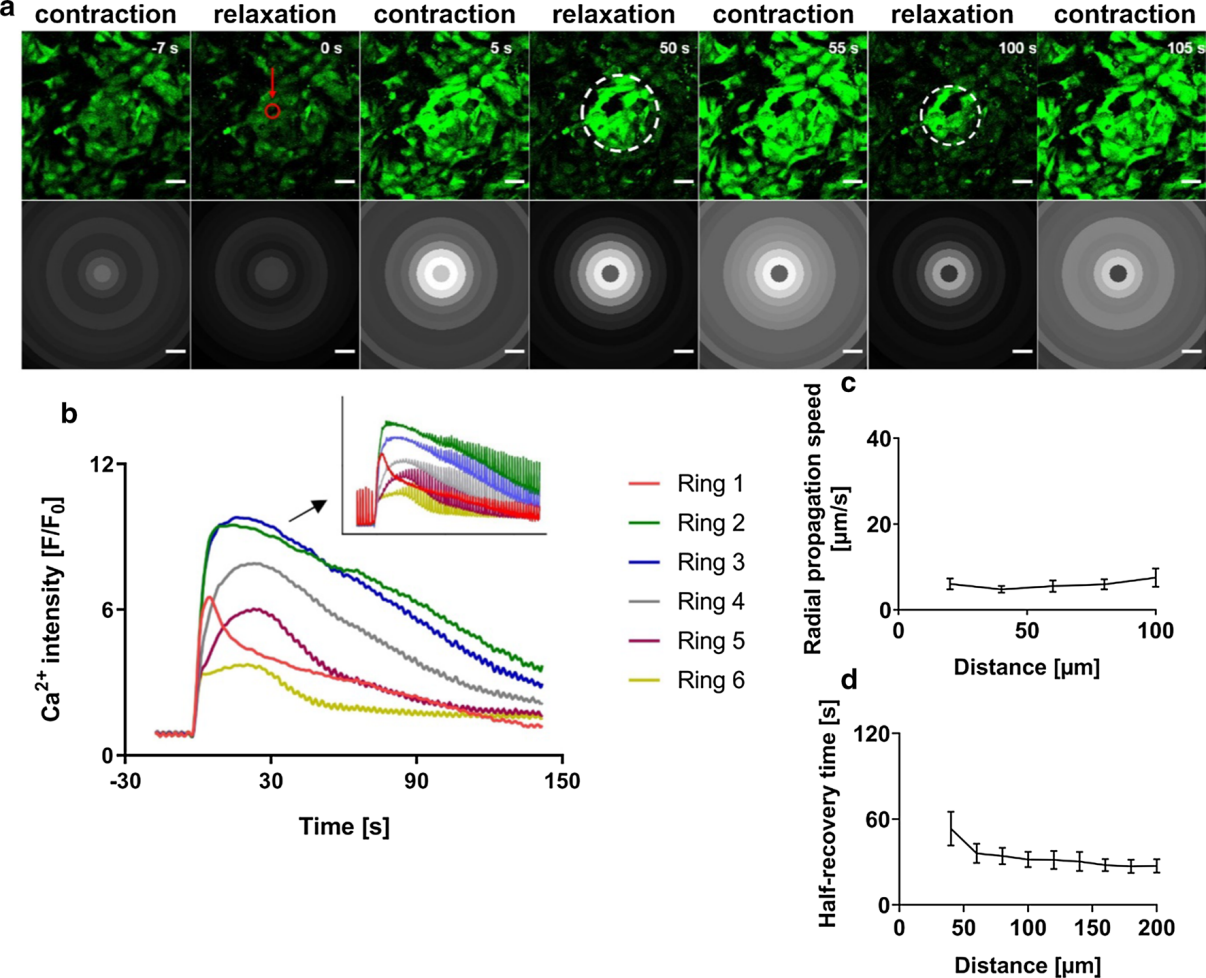
Laser-induced cardiomyocyte death resulted in a temporal intracellular calcium-increase in surrounding cardiomyocytes. **a** Time-series images showing calcium waves in fluorescently labelled contracting myocytes (green) representing contracted and relaxed states, before and after ablation, with the ablated cell (red) in the central ring. Time-point of ablation is set to t = 0 s, refer SAdditional file 1: movie 1. Corresponding grey-scale images show average grey values over each of the concentric rings (Ring 1–6) for that time point. The dashed ring at time point 50 s and 100 s indicates the decreasing affected area for high [Ca^2+^]_i_ with time. Scale bars: 50 µm. **b** Changes in Ca^2+^ fluorescence intensity (i.e. the slowly varying component of fluorescence signals averaged over each ring) with time, indicating [Ca^2+^]_i_ of cells before and after ablation. Inlay indicates original Ca^2+^ fluorescence intensity values plotted against time with the same scale for both axes as in the main plot. **c** Mean radial Ca^2+^ propagation speed after ablation (see section Methods) across distance of up to 100 µm (Ring 1–5) from the ablated cell (R0), with error bars showing s.e.m., N = 10*.* **d** Mean time of recovery of contractility in cells (50% of amplitude before ablation, see section Methods) after ablation, across a distance of 200 µm (Ring 1–10) from the ablated cell, with error bars showing s.e.m., N = 10

To investigate the effect of single-cell death, the coculture systems described above were used. Cultured myocardial cells contracted rhythmically and synchronously in clusters from 48 to 72 h. Periodic Ca^2+^ waves were observed in these cells as a result of local cytosolic Ca^2+^ increase. In manually selected cell clusters containing numerous myocytes interspersed with some non-myocytes, contracted and relaxed states of the myocytes could be well distinguished by the Ca^2+^ signal (as shown in Fig. 3a). Laser irradiation of a single target myocyte (indicated in red in Fig. 3a and Additional file 1: movie 1) resulted in mechanical disruption and eventual death of the cell as seen from the gradual depletion of Ca^2+^ intensity in the ablated cell. With time, however, death of a cardiomyocyte resulted in a strong increase in intracellular calcium concentration ([Ca^2+^]_i_) in the neighboring CMs of the cluster. Fluo-4AM intensity analysis confirmed the sharp increase for surrounding rings (Fig. 3b). Ca^2+^ fluorescence-intensity changes were plotted as F/F_0_ ratio, where F represent the fluorescence intensity (slowly varying component) at any given time point during the experimental time-frame and F0 represents the average Ca^2+^ intensity before ablation determined from the slowly varying signal component. Increased cytosolic Ca^2+^ resulted in impaired contractility of the cells as seen from the lack of distinct calcium peaks briefly after ablation. Interestingly, increased [Ca^2+^]_i_ after ablation recovered to normal levels with time. This effect went along with renewed detection of spontaneous Ca^2+^ oscillations and contractility.

We observed that the magnitude of [Ca^2+^]_i_ increase was higher in rings closer to the ablated cell and decreased with distance (Fig. 3b). Following the time-point of maximum intensity (t_[Ca2+]max_) for each ring, we found a time delay for maximum intensity between one ring and the next ring, suggesting that Ca^2+^ propagated along the successive rings starting from the point of ablation. The radial Ca^2+^ propagation speed across each ring was calculated with speeds in the order of µm/s, ranging from 7 µm/s (s.e.m. 2 µm/s) to 4 µm/s (s.e.m. 0.7 µm/s) (Fig. 3c) for N = 10 (where N refers to individual isolates; sample size including replicates, n = 15).

Quantifying the recovery time for newly established Ca^2+^ oscillations, for most experiments no complete recovery was observed in many of the inner analysis rings (marked by dashed white ring in Fig. 3a) during the experimental time-frame. Hence, the time for partial recovery (50% of initial amplitude) was determined and plotted as mean half-recovery time for all rings (Fig. 3d). Obviously, the farther the distance from the point of ablation, the faster the cells recovered, with half-recovery time ranging from 53 s (s.e.m. 11 s) in the immediate ring (Ring 2) to 27 s (s.e.m. 4 s) in the last ring (Ring 10) for N = 10 (n = 14). Ablation of CM in cocultures, therefore, triggered Ca^2+^ propagation to connected CMs of the cluster resulting in impaired CM function, that is, loss of contractility. Thus contractility recovered with gradual decrease in [Ca^2+^]_i_ over time from the outside towards the point of ablation.

### Ablation results in increased [Ca^2+^] in surrounding cells of cardiomyocytes-enriched cell cultures

**Fig. 4:**
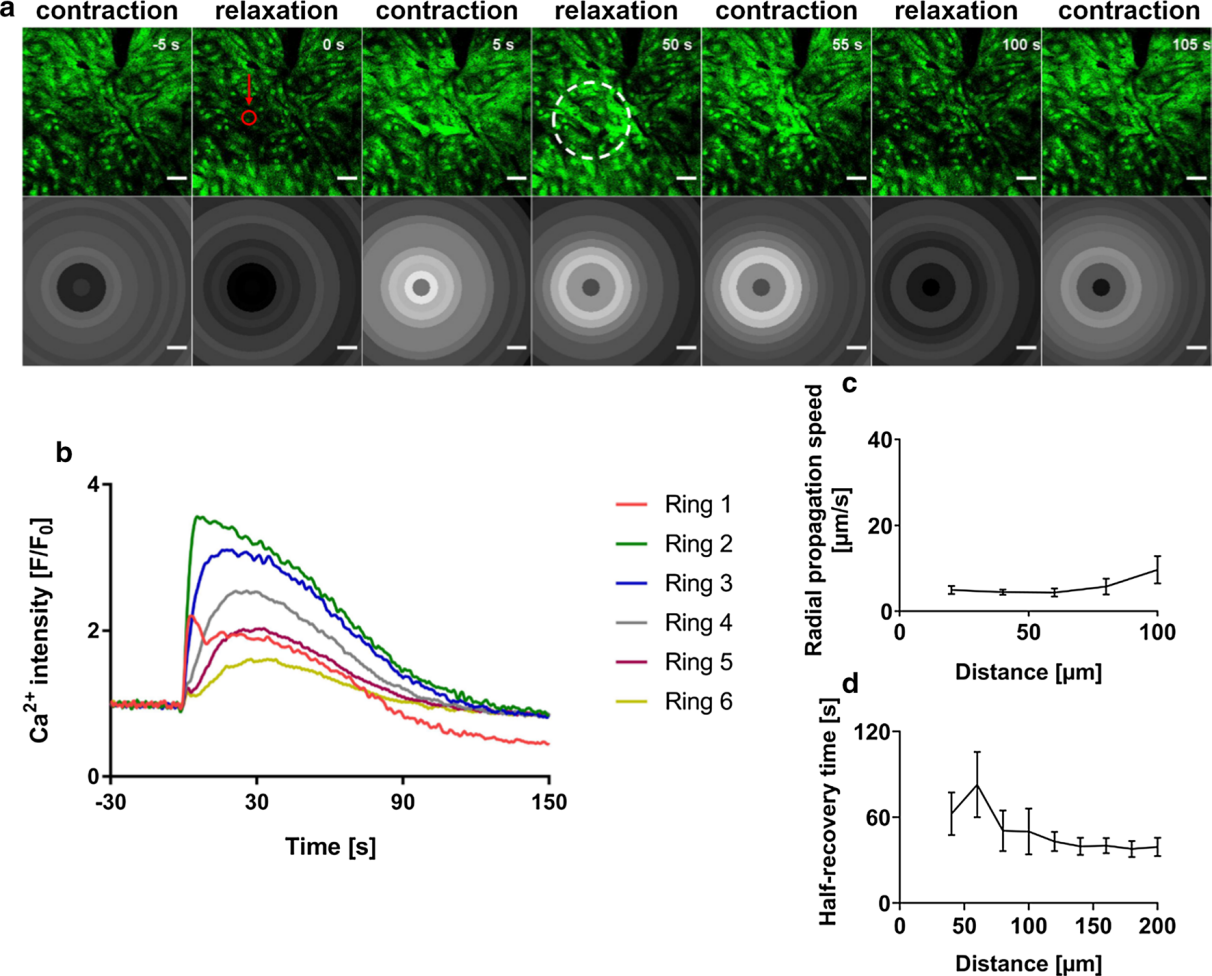
Impact of cardiomyocyte death in cardiomyocytes-enriched cultures. **a** Time-series images (green) showing calcium waves in fluorescently labelled contracting myocytes representing contracted and relaxed states, before and after ablation. Ablated cell is indicated by a red circle. Times before and after ablation (t = 0) are indicated. Corresponding grey-scale images show grey values averaged over each of the concentric rings (Ring 1–6) at that time point. The dashed ring at time point 50 s indicates the affected area for high [Ca^2+^]_i_. After additional 50 s full recovery took place. Scale bars: 50 µm. **b** Changes in Ca^2+^ with time based on the values from the slowly varying component of fluorescence intensity. Each Ca^2+^ peak represents contraction and relaxation events of all cells in the respective ring. **c** Mean Ca^2+^ propagation speed in myocytes after ablation across a distance of 100 µm (Ring 1–5), with error bars showing s.e.m, N = 6. **d** Mean time of recovery of contractility in cells (50% of amplitude before ablation) after ablation across a distance of 200 µm (Ring 1–10) from ablation point (R0) with error bars showing s.e.m., N = 6

In order to study the impact of single-cell death on a homogeneous cell population, we performed the same experimental procedure in myocytes-enriched cultures. Cell clusters with predominantly contracting myocytes were chosen for ablation. Laser-induced death of CM in these clusters resulted in increased [Ca^2+^]_i_ in surrounding CMs as shown before (Fig. 4a). [Ca^2+^]_i_ changes were seen upon ablation from the fluorescence intensity as seen in Fig. 4b. Interestingly, Ca^2+^ recovery was more heterogeneous than in mixed cultures and decreased completely to basal levels in some experiments over time (Fig. 4b). Ca^2+^ propagation speed in myocytes-enriched culture ranged between 9 µm/s (s.e.m. 3 µm/s) and 4 µm/s (s.e.m. 0.9 µm/s) for N = 6 (n = 11), as shown in Fig. 4 c. Similarly, as in coculture, cells in the farther rings recovered faster with a half-recovery time of 39 s (s.e.m. 6 s) (in Ring 10) compared to the cells in closer rings, with a half-recovery time of 62 s (s.e.m. 14 s) (in Ring 2) for N = 6 (n = 9) as seen in Fig. 4d.

### Characterization of impact of laser ablation on non-myocytes in cocultures

**Fig. 5:**
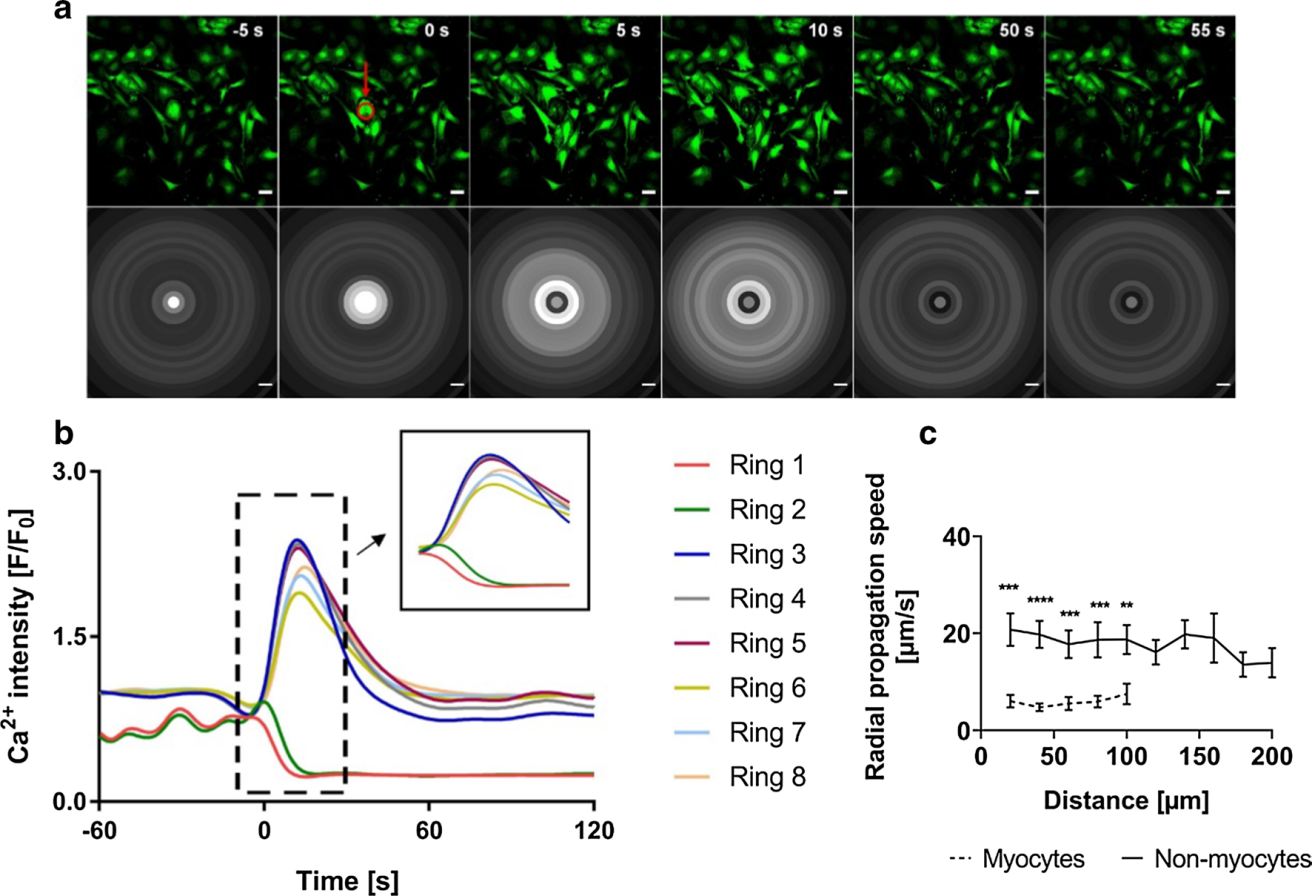
Impact of ablation-induced cell death on non-myocytes in cocultures. **a** Time-series images (green) showing propagation of a solitary, rapid transient wave of Ca^2+^ along the cells after ablation of a central myocyte (red, refer Additional file 2: movie 2). Corresponding grey-scale images show grey values averaged over each of the concentric rings at that time point. Time-point of ablation is set to t = 0 s. Scale bars: 50 µm. **b** Plot depicting changes in Ca^2+^ fluorescence intensity with time, indicating averaged [Ca^2+^]_i_ of rings (Ring 1–8) before and after ablation. Inlay shows a zoom-in of the single Ca^2+^ spike moving sequentially through the subsequent rings. **c** Mean Ca^2+^ propagation speed along the myocytes (dotted) and non-myocytes across the rings (Ring 1–10) after ablation in cocultures, with error bars showing s.e.m., N = 10 for myocytes, N = 8 for non-myocytes*.* The propagation speeds were significantly higher along the non-myocytes compared to those in myocytes for a distance of 100 µm from the ablated cell (Multiple t-tests, Holm-Šídák method, α = 0.05)

To characterize the impact of ablation on non-myocyte cells, we ablated single cells (myocyte/non-myocyte) in cocultures and observed the effect particularly pronounced for surrounding non-myocytes. Cell clusters were chosen such that the central target cells were surrounded by numerous non-myocytes on all sides. In contrast to the briefly sustained [Ca^2+^]_i_ increase in beating cardiomyocytes, we observed upon ablation a single, transient Ca^2+^ spike in non-myocytes that was propagating like a solitary wave front through the surrounding non-myocytes (Fig. 5a and Additional file 2: movie 2). This Ca^2+^ spike formation was induced irrespective of whether myocytes or non-myocytes were ablated. Analyzing the Ca^2+^-changes with time showed for non-myocyte cells a momentary increase in [Ca^2+^]_i_ after ablation that was followed by a drop in intensity to values that were identical to the Ca^2+^ levels before ablation. Further, the magnitude of increase in [Ca^2+^]_i_ was relatively lower in non-myocytes as compared to the increase in myocytes. Ca^2+^ propagation speeds were calculated across a distance of up to 200 µm from the ablated cell with almost constant values of around 20 µm/s independent of distance to the point of ablation (N = 8). Such values were at least three-fold higher and significantly different when compared to the propagation speed in myocytes (Fig. 5c; Multiple t-tests, Holm-Šídák method, α = 0.05).

### Ca^2+^ propagation triggered by ablation follows a completely different pattern in cardiac myocytes and non-myocytes

**Fig. 6:**
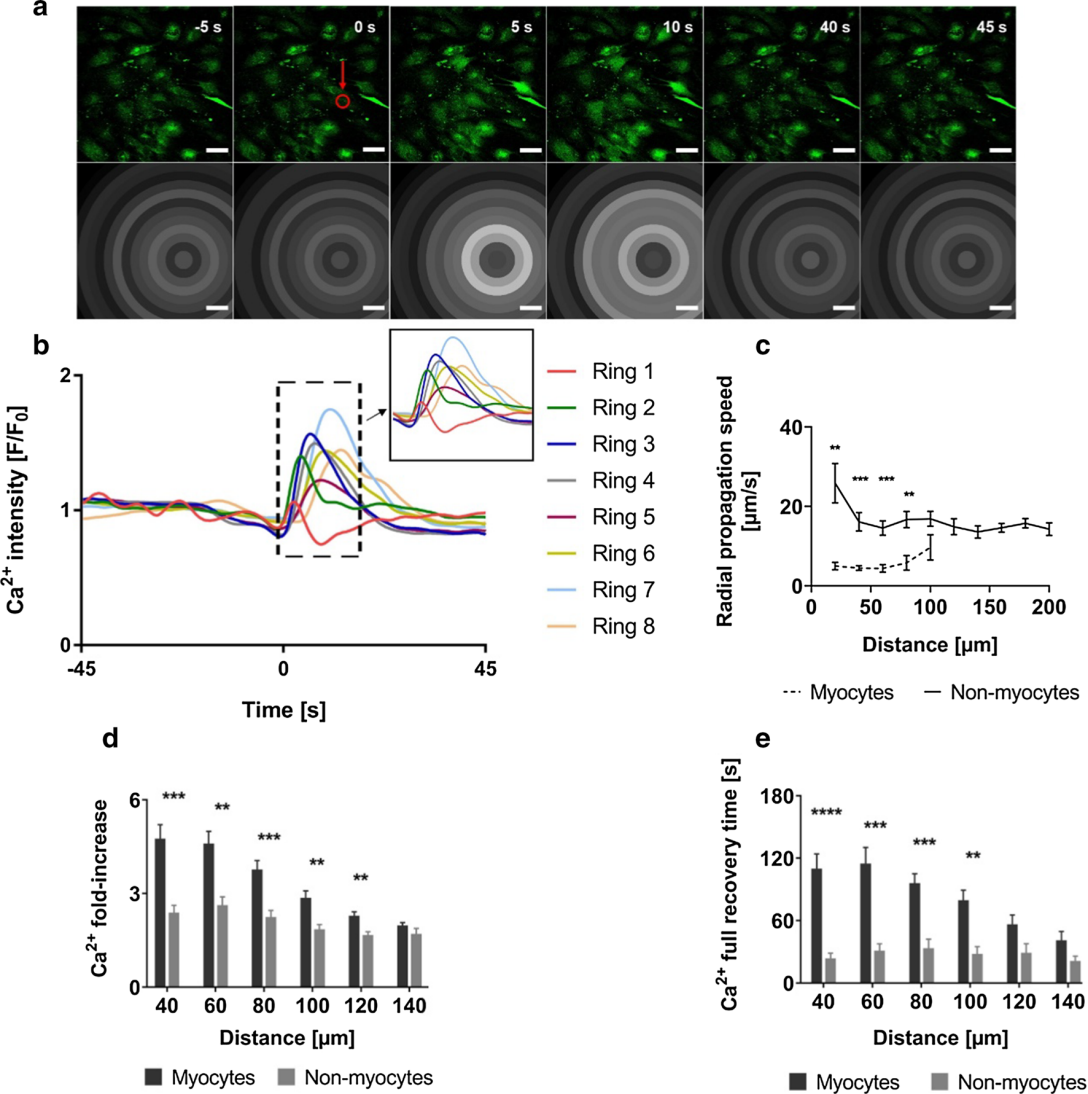
Ablation of fibroblasts in myocytes-depleted cultures. **a** Time-series images (green) showing propagation of a transient, solitary wave of Ca^2+^ along fluorescently labeled fibroblasts after single-fibroblast ablation (red, refer Additional file 3: movie 3). Corresponding grey-scale images show grey values averaged over each of the concentric rings (Ring 1–8) for that time point. Scale bars: 50 µm. **b** Plot depicting changes in Ca^2+^ fluorescence intensity with time, indicating [Ca^2+^]_i_ of cells before and after ablation. Inlay shows a zoom-in of the Ca^2+^ spike moving sequentially along the subsequent rings. **c** Mean Ca^2+^ propagation speed across the rings (Ring 1–10) after ablation in myocytes from myocyte-enriched cultures (dotted) and non-myocytes from myocyte-depleted cultures, with error bars showing s.e.m., N = 6 for myocytes; N = 7 for non-myocytes*.* The propagation speeds were significantly higher along the non-myocytes compared to those in myocytes for a distance of 100 µm from the ablated cell (Multiple t-tests, Holm-Šídák method, α = 0.05). **d** Plot depicting mean fold-increase in [Ca^2+^]_i_ after ablation in myocytes and non-myocytes across all culture systems over a distance of 100 µm from the ablated cell, with error bars showing s.e.m., N = 16 for myocytes; N = 15 for non-myocytes. **e** Plot depicting mean Ca^2+^ full recovery times in myocytes and non-myocytes across all culture systems over a distance of 100 µm from the ablated cell, with error bars showing s.e.m., N = 14 for myocytes; N = 13 for non-myocytes. Both Ca^2+^ fold-increase as well as the Ca^2+^ full recovery times were significantly higher in myocytes than in non-myocytes across cocultures and enriched/depleted cultures for a distance of 100 µm from the ablated cell (Multiple t-tests, Holm-Šídák method, α = 0.05)

Since fibroblasts constitute a major proportion of the non-myocyte population, we next investigated if the effects of ablation in non-myocytes were also present in myocyte-depleted cultures. Although some samples of myocytes-depleted cultures still contained numerous myocytes, several clusters of non-myocytes could be always found. Thereby, cell clusters with predominantly non-myocytes were chosen in these samples. Upon single cell ablation we observed the same single, transient Ca^2+^ spike propagating like a solitary wave front through the cells in all directions (Fig. 6 a, b and Additional file 3: movie 3) just as observed in coculture non-myocytes. With approximately 20 µm/s also the Ca^2+^ propagation speed across all the selected rings showed a similarly fast and not decreasing speed with distance as found before (N = 7, n = 14). As before, also this speed was significantly higher when compared to myocytes (Fig. 6c). [Ca^2+^]_i_ fold-change analyses clearly showed for all culture systems, a clear increase in [Ca^2+^]_i_ after ablation. This increase was significantly higher in myocytes (N = 16, n = 26) than in non-myocytes (N = 15, n = 22) (Fig. 6d, Multiple t-tests, Holm-Šídák method, α = 0.05). Moreover, [Ca^2+^]_i_ full recovery behavior was also found to be different in myocytes and non-myocytes across all culture systems. Comparison of [Ca^2+^]_i_ full recovery times in myocytes (N = 14, n = 20) and non-myocytes (N = 13, n = 19) showed significantly faster recovery in non-myocytes over the complete distance of 100 µm from the ablated cell as seen in Fig. 6 e (Multiple t-tests, Holm-Šídák method, α = 0.05). Furthermore, [Ca^2+^]_i_ fold-increase as well as recovery time were largely independent on distance to the ablated cell for non-myocyte cultures. In contrast, for myocytes a clear decreasing effect was present with distance. Thereby, the clear difference between the above detailed outcomes of ablation argues for a different mechanism underlying Ca^2+^ propagation and regulation in cardiomyocytes and fibroblasts.

### Gap junctions could be critical regulators of Ca^2+^ propagation in cardiomyocytes upon laser induced single-cell death

**Fig. 7:**
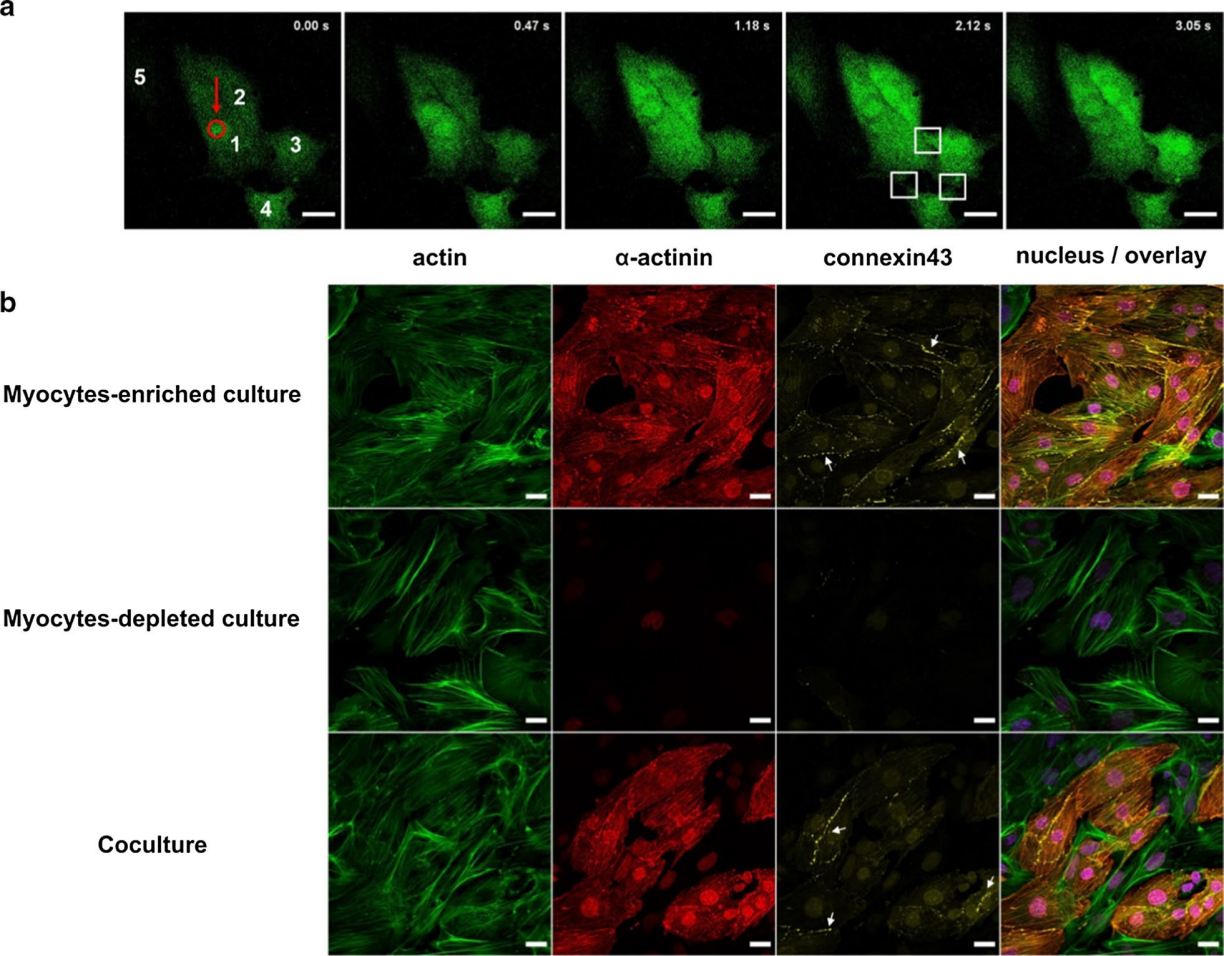
Intercellular Ca^2+^ diffusion at cell–cell junctions in cardiomyocytes. **a** Time-series images showing Ca^2+^ propagation along Fluo-4 labeled cardiomyocytes (green) after ablation (refer Additional file 4: movie 4). Ablated spot is indicated in red. The connected cells in the cluster are numbered 1–4 and the connections are marked by white squares. Scale bars: 20 µm*.* **b** Immunocytochemical analysis revealed the presence of gap junction protein Cx43 (yellow) at cell–cell contacts (indicated by arrows) between cardiomyocytes from coculture as well as myocytes-enriched culture. The cells were stained for myocyte-specific α-actinin (red), actin filaments (green) and nuclei (blue). Cx43 was absent between myocytes and fibroblasts as well as between fibroblasts in coculture and myocytes-depleted culture. Scale bars: 10 µm

To understand the mechanism underlying Ca^2+^ propagation in cardiomyocytes in more detail, clusters of three to four contracting cardiomyocytes were chosen from an enriched myocytes culture. Ablation of a single cardiomyocyte confirmed the increase in [Ca^2+^] in connected cells over time. Following the Ca^2+^ changes in cells with high temporal resolution, we observed the flow of Ca^2+^ from one cell to another in a sequential manner (Additional file 4: movie 4) starting from distant points at the cell borders. Ca^2+^ propagation was completed after approximately 2 s (Fig. 7a). Since intercellular Ca^2+^ signaling in cardiomyocytes is mediated by gap junction channels, we checked for the presence of gap junction channel protein connexin43 (Cx43) in cocultures as well as enriched cultures. Immunocytochemical analysis confirmed the localization of Cx43 in cardiomyocytes at the cellular periphery where intercellular junctions are formed while being absent in CM-Fb and Fb-Fb cell–cell contacts (Fig. 7b). Cx43 plaques between myocytes were observed both in cocultures and myocytes-enriched cultures.

## Discussion

Cell death has been found to be a hallmark characteristic of various cardiac pathologies such as heart failure, myocardial infarct and ischemia/reperfusion [34]. Although apoptotic, necrotic and autophagic regulation of CM death under stress have been described so far [8], the spontaneous signals following CMs death and its response in connected myocardial cells are still incompletely understood but could provide a detailed understanding of stress-induced signaling and functional interconnectivity in these cells [35, 36]. Our results clearly showed that single-cell ablation triggered Ca^2+^-signal propagation in both CMs and Fbs. In CMs, influx of Ca^2+^ upon ablation resulted in impaired beating as the cells remained in a contracted state for a prolonged period until excess Ca^2+^ was cleared from the cell. Contraction of cells was restored gradually with decrease in [Ca^2+^]_i_ as seen from the reappearance of distinct Ca^2+^ peaks. Importantly, the decreasing magnitude of Ca^2+^-influx in successive rings indicates that this ablation-induced Ca^2+^-propagation in CMs is distance-dependent. Further, sequential diffusion of Ca^2+^ through cell–cell contacts (Additional file 4: movie 4) and Cx43 localization at intercellular CM junctions argue for a gap junction dependent Ca^2+^ flow [37, 38]. Interestingly, efflux of excess cytosolic Ca^2+^ was required for further contractions in CMs. Slower recovery of cells located close to the ablated cell compared to distant cells argues for higher Ca^2+^ influx at sites of ablation and indicates a continuously active, systemic Ca^2+^ efflux machinery [27] that decreases [Ca^2+^]_i_ with distance. In CMs, efflux of excess cytosolic Ca^2+^ is regulated predominantly by sodium-calcium exchanger (NCX) and sarcoplasmic reticulum Ca^2+^ ATPase (SERCA) pumps [39]. In addition, decreasing [Ca^2+^]_i_ of the same cells with time must depend on an adaptation mechanism that either activates Ca^2+^ efflux rates of underlying transport proteins in a time range of seconds or regulates Ca^2+^ flow through gap junctions. Especially for the latter, gating mechanisms have been identified that can cause uncoupling of GJs at high [Ca^2+^]_i_ [40]. Reduced [Ca^2+^]_i_ additionally allowed recovery of contractility as indicated by regained synchronized Ca^2+^ spikes. Only for some cells [Ca^2+^]_i_ remained high to result in retraction of membranes after ablation (Additional file 5: movie 5). These cells may have been permanently/irreversibly impaired due to excess cytosolic Ca^2+^ and might confirm results showing that cytosolic Ca^2+^ overload is detrimental to CMs. Ca^2+^ overload has been shown to have electrical (arrhythmia caused by oscillatory potential) as well as mechanical (increased contracture, decreased contractile forces and aftercontractions) manifestations in the heart. Moreover, increased [Ca^2+^]_i_ triggers signaling cascades leading to calpain-mediated proteolysis, hypertrophy and mitochondria-mediated necrotic death of CMs [41–43].

Ca^2+^ propagation speeds between myocytes were comparable in coculture and cardiomyocytes-enriched systems and similar to values reported in previous studies [44]. Interestingly, faster contractile half-recovery in most of the rings in cocultures than in CM-enriched cultures (cf. Figs. 3d and 4d) suggests that the presence of fibroblasts may influence cellular responses upon massive Ca^2+^ inflow. Further, Fb–Fb Ca^2+^ propagation speeds were clearly higher than in CMs but comparable in cocultures and myocyte-depleted systems. These findings argue for a different Ca^2+^ signaling/transport mechanisms between CM–CM, CM–Fb and Fb–Fb pairs. Immunocytochemical analysis identified Cx43, the main component of mammalian gap junctions [45] in CMs. Of note, we did not observe gap junctions, which facilitate flow of Ca^2+^ and other molecules up to a size of 1 kDa across cells [38], in our experiments on cultured embryonic Fbs. Previous research has shown the presence of connexin-based contacts in CM–Fb and Fb–Fb cell pairs both in tissues (ventricular myocardium of adult rat and infarcted hearts of adult sheep) as well as cultured cells (neonatal rat hearts) [21, 46]. However, regulation of functional connectivity in these cell pairs by connexin-based GJs is not clearly understood. CM and Fb connectivity was shown to be mediated also by tunneling nanotubes both in vitro and in vivo [47]. These structures were shown to mediate Ca^2+^ fluxes across cells in other cell types [48, 49]. In this respect, transient receptor potential (TRP) channels might facilitate the non-selective entry of cations such as Ca^2+^ into Fbs [50, 51] and could represent a possible mechanism for Ca^2+^ signaling in fibroblasts. Moreover, TRPM-7 mediated Ca^2+^ signals have been shown to play an essential role for atrial fibroblasts leading to fibrosis that could eventually result in atrial fibrillation [52]. Most interestingly, although not gated by voltage, latest studies could show, that TRP channels can also cause membrane depolarization to indirectly influence Ca^2+^ influx via other Ca^2+^-permeable channels. Here, store-operated calcium channels (SOC) [53, 54] or STIM/ORAI channels [55] might play an important role. Furthermore, TRP channel activity can be influenced by membrane potentials [56]. Although the voltage-gated L-type channels that regulate Ca^2+^ entry in human CMs are absent in Fbs [53, 57], studies have shown evidence that functional voltage-gated ion channels are expressed in cardiac fibroblasts [58].

This growing number of studies could form the basis for understanding the different Ca^2+^ handling in CMs and Fbs. Although the exact mechanism of Ca^2+^ signal propagation in CM–Fb and Fb–Fb pairs is still unclear, our findings identify differential Ca^2+^-handling and propagation in CMs and Fbs. Ca^2+^ signal propagation speeds and high resolution analysis performed here argue for a most likely diffusion driven propagation in CMs and a putative membrane potential dependent propagation in Fbs. Since Ca^2+^ signals are involved in cell death signaling pathways in CMs, investigating regulation of Ca^2+^ in myocardial cells may provide a potential target pathway in cardiac diseases [8, 59]. Moreover, as Fbs have been shown to be involved in impulse propagation and electrical coupling of CMs separated by long distances [60], further research on Ca^2+^ signaling in cardiac Fbs and their interactions with CMs is needed to understand the role of Fbs in normal and diseased myocardium. Analysis of long-term effects of ablation-induced Ca^2+^ changes on CM functionality such as beat frequency, cytoskeletal adaptations, and contractile force as well as membrane potential-dependent Ca^2+^ changes in fibroblasts will be furthermore important to characterize the overall characteristics of single-cell death in myocardial tissues.

## Conclusions

Laser-induced death of single myocardial cells resulted in Ca^2+^ propagation from the ablated cell to connected cells in cell clusters. Characterization of this laser-induced Ca^2+^ propagation showed a different propagation pattern in cardiomyocytes (CMs) and fibroblasts (Fbs). In CMs, the Ca^2+^ propagation was slow and briefly sustained while in fibroblasts the Ca^2+^ propagated as a solitary, rapid transient wave. Increased intracellular Ca^2+^ concentration in CMs resulted in temporary loss of contractions. However, with decreasing [Ca^2+^]_i_ the contractility reappeared. The difference in Ca^2+^ propagation in CMs and Fbs indicated a difference in Ca^2+^ handling in these cell types. Since dysregulation of [Ca^2+^]_i_ have been found underlying several heart pathologies, understanding these mechanisms could provide valuable knowledge on cell death/survival signaling pathways in myocardial cells.

## Supplementary Information

**Additional file 1: Movie 1**. Ablation-induced Ca^2+^ propagation in coculture myocytes: Time series depicting Ca^2+^ propagating along the myocytes upon ablation of a single myocyte. The ablated myocyte is indicated by red circle. An increase in Ca^2+^ can be seen in connected myocytes after ablation (t = 0 s). Distant cells are little or not affected by ablation, as indicated by continuing Ca^2+^ waves even after ablation. Ca^2+^ intensity values of closely connected myocytes return to values identical to intensity values before ablation.

**Additional file 2: Movie 2**. Ablation-induced Ca^2+^ propagation in non-myocytes in cocultures: Time-series depicting Ca^2+^ propagating along the non-myocytes upon ablation of single-central myocyte. The ablated myocyte is indicated by red circle. A spike in Ca^2+^ can be seen after ablation (t = 0 s), after which the Ca^2+^ intensity drops to values values identical to intensity before ablation.

**Additional file 3: Movie 3**. Ablation-induced Ca^2+^ propagation in non-myocytes in myocytes-depleted cultures: Time-series depicting Ca^2+^ propagating along non-myocytes upon ablation of a single non-myocyte in the cluster. The ablated cell is indicated by a red circle. A spike in Ca^2+^ can be seen after ablation (t = 0 s), after which the Ca^2+^ intensity drops to values that are identical to the intensity before ablation.

**Additional file 4: Movie 4**. Time-series showing sequential diffusion of Ca^2+^ across connected cardiomyocytes: Ablation-induced diffusion of Ca^2+^ across Fluo-4 labelled cardiomyocytes through cell-cell contacts. Ablated region is indicated by red circle and the time point of ablation is set to t = 0 s. Ca^2+^ sequentially diffuses across the three connected cells in about 2 s from the time point of ablation. The closely connected myocyte exhibits rapid intracellular Ca^2+^ oscillations with time.

**Additional file 5: Movie 5**. Ablation-induced changes in surrounding cardiomyocytes in cluster: [Ca^2+^]_i_-increase in connected cells after ablation is accompained by membrane retraction in connected cells of the cluster. The ablated region is indicated by red circle and the retraction of cells in the cluster is indicated by arrows. The time point of ablation is set as t = 0 s.

## Data Availability

All datasets used and/or analysed during the current study are available from the corresponding author on reasonable request.All datasets used and/or analysed during the current study are available from the corresponding author on reasonable request.

## Abbreviations

biotin-DOPE: 1,2-Dioleoyl-sn-glycero-3-phosphoethanolamine-*N*-(cap biotinyl)
CB: Cytoskeletal buffer
CICR: Calcium-induced calcium release
CM: Cardiomyocyte
Cx40: Connexin40
Cx43: Connexin43
Cx45: Connexin45
DiR: 1,10-Dioctadecyl-3,3,30,30-tetramethylindotricarbocyanine iodide
DMEM: Dulbecco’s minimum essential medium
DOPE: 1,2-Dioleoyl-sn-glycero-3-phosphoethanolamine
DOTAP: 1,2-Dioleoyl-3-trimethylammonium-propane
EBSS: Earle’s balanced salt solution
ECM: Extracellular matrix
EGTA: Ethylene glycol-bis(2-aminoethylether)-*N*,*N*,*N*′,*N*′-tetraacetic acid
Fb: Fibroblast
FLs: Fusogenic liposomes
GJ: Gap Junction
LSM: Laser scanning microscope
MES: 2-(4-Morpholino)ethanesulfonic acid
MI: Myocardial infarction
NA: Numerical aperture
NCX: Sodium–Calcium exchanger
PBS: Phosphate buffered saline
PMCA: Plasma Membrane Calcium ATPase
px: Pixel
RT: Room temperature
RyRs: Ryanodine receptors
SERCA: Sarcoplasmic/Endoplasmic reticulum Calcium ATPase
SOC: Store operated channels
TRP: Transient receptor potential
